# Human Information Processing of the Speed of Various Movements Estimated Based on Trajectory Change

**DOI:** 10.3390/e25040695

**Published:** 2023-04-20

**Authors:** Hiroki Murakami, Norimasa Yamada

**Affiliations:** Graduate School of Health and Sport Sciences, Chukyo University, 101 Tokodachi, Kaizu-cho, Toyota, Aichi 470-0393, Japan

**Keywords:** Fitts, Woodworth, information entropy, mutual information

## Abstract

Fitts’ approach, which examines the information processing of the human motor system, has the problem that the movement speed is controlled by the difficulty index of the task, which the participant uniquely sets, but it is an arbitrary speed. This study rigorously aims to examine the relationship between movement speed and information processing using Woodworth’s method to control movement speed. Furthermore, we examined movement information processing using an approach that calculates probability-based information entropy and mutual information quantity between points from trajectory analysis. Overall, 17 experimental conditions were applied, 16 being externally controlled and one being self-paced with maximum speed. Considering that information processing occurs when irregularities decrease, the point at which information processing occurs switches at a movement frequency of approximately 3.0–3.25 Hz. Previous findings have suggested that motor control switches with increasing movement speed; thus, our approach helps explore human information processing in detail. Note that the characteristics of information processing in movement speed changes that were identified in this study were derived from one participant, but they are important characteristics of human motor control.

## 1. Introduction

The relationship between the speed and accuracy of human movement, relevant to the human performance theory systematized by Fitts and Posner [1], was first investigated by Woodworth [2]. In a simple line-drawing task, he examined the relationship between the error from a target point and the speed of motion at which the participant moved a fixed distance at various speeds. Later, Fitts [3,4], known for his famous experiments, established an index of difficulty (ID) determined from the distance and accuracy requirements of the exercise and described a relationship whereby the higher this index, the longer the corresponding movement time (MT). This is widely known as “Fitts’ Law” and the “speed and accuracy trade-off.” Furthermore, Fitts expressed this index of difficulty (ID) in terms of units of information (bits) and considered that the increase in MT was related to information processing, corresponding to the amount of information. This was also an attempt to generalize human movement using Shannon’s information theory [5], a mainstream theme of the time. This theory is an extension of Hartley’s study [6], which defined information as “a set of symbols obtained by successive choices (including elimination)” and contributed to the development of information theory. After Shannon’s information theory was published, it was adapted to psychology [7,8,9] and developed into an analogy to understand the human mind as a computer that processes information. Moreover, it developed into a theory of human information processing. Fitts adapted this theory to the relationship between the speed and accuracy of the human movement systems that Woodworth discovered and linked to information. “Fitts’ law” is recognized as the most crucial basic principle of human physical movement [10]. However, the information processing of human movements, as determined by Fitts, is indicated by a unique value for the average amount of information per unit of time. The type of processing that occurs remains unclear. Fitts’ experiment showed that the formalized ID value controlled movement speed approximately but not precisely. This can be understood from Fitts’s statement [3]: “...However, the recording and measurement of successive movement amplitudes are laborious. Fortunately, for the present purpose, a man can adjust his average performance rate to meet the demands of a particular task, such as the requirement that his movements fall within a specified tolerance range...” In other words, Fitts designed the experiment to maintain a constant average movement speed. However, participants’ arbitrary speed determined the movement’s speed. Specifically, in Fitts’ experiment, an extension of Woodworth’s experiment, which dealt with motion in a two-dimensional plane to motion in a three-dimensional space, the speed of the motion was not strictly controlled.

Several studies have focused on the variations in trajectories during motion [11,12]. In particular, our previous studies [13,14] focused on the trajectory variation process of motion during Fitts’ experiments. This is an extension of our previous studies, which focused on endpoint fluctuations of trajectories, quantified them, and examined information processing using an approach that focused on periodic fluctuations of movements. We are currently beginning to understand that the amount of human movement, examined only from the average amount of information, differs at each point in the trajectory. The point at which processing takes place varies slightly depending on the difficulty. Following the basic concept of Shannon’s information theory [5], we theorize that if the amount of information (magnitude of variation) moving from point A to point B decreases, it can be viewed as a decrease in irregularity; thus, further processing (information processing) occurs. In particular, recent results have shown that the amount of information at the midpoint of the trajectory and the target point shows a characteristic change with the magnitude of the velocity of different IDs. However, this experiment only compared two IDs. Several problems exist regarding examining the differences in IDs, such as the ones described above and determining which information processing is appropriate for each control style (feedforward and feedback). The study by Murakami and Yamada [14] did not consider this issue in depth. To further examine the characteristics of information processing associated with the speed of movement, it is necessary to control the speed of movement externally, as in Woodworth’s experiment.

Therefore, this study aimed to investigate the relationship between the speed of movement and information processing by introducing Woodworth’s method of externally controlling the speed of movement into Fitts’ experiment, tapping the exact distance between targets at different speeds, and quantifying the trajectory change process. It has been pointed out that the trajectories of hand movements, such as those in this study, vary among individuals [15]. Therefore, it was first necessary to examine each participant’s characteristics individually. Thus, as a first step before examining the generality of the relationship between speed and information processing from the data of many participants, we used a previous method [14], which examined several trials by a single participant.

## 2. Materials and Methods

### 2.1. Participant

One healthy right-handed man (age: 25) enrolled in the researchers’ university participated in the study. The participant self-reported having normal vision and no motor impairments. All study procedures were conducted per the Declaration of Helsinki and the ethics code of Chukyo University and were approved by the ethics committee of Chukyo University (approval number: 2018–029). The participant provided written informed consent before participation.

### 2.2. Apparatus

**Figure 1:**
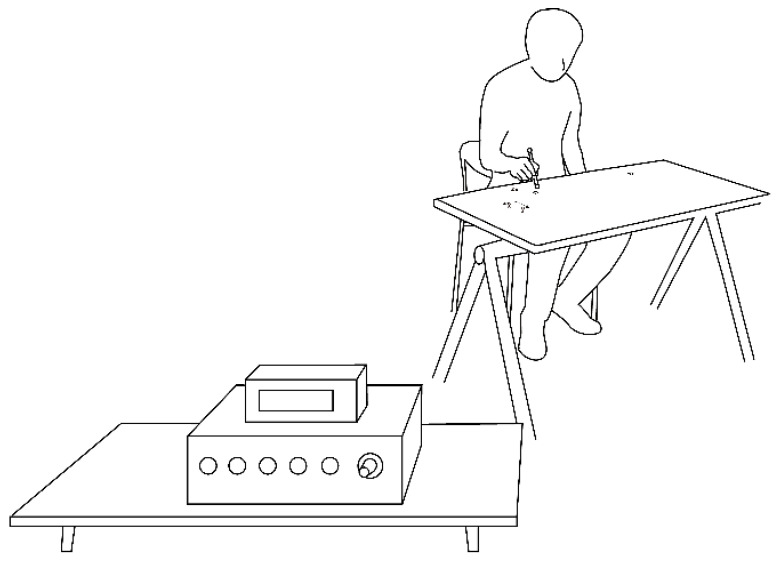
Conceptual diagram of the experiment setup. In the frequency conditions (see Section 2.3), the trials were matched to the auditory signal supplied by the frequency oscillator in the figure.

A motion capture system (MAC3D System, Motion Analysis Corporation, Rohnert Park, CA, USA, 200 Hz) with six 300,000-pixel cameras arranged around the participant was controlled by software (Cortex version 7, Motion Analysis Corporation, USA) to acquire the coordinates of the reflection markers attached to the pen tip. Three-dimensional axes were set in the software, with the *x*-axis pointing toward the distance between targets (Figure 1). Additionally, the software synchronized the analog signal of the frequency oscillator supplying the auditory signal and the motion capture system. That analog signal was also acquired at 200 Hz. Based on the calibration performed before the experiment, the accuracy of the analysis was 0.01 ± 0.63 mm of residual from the reference value in the space of 1.8 × 1.8 × 1.8 (m^3^).

### 2.3. Experimental Design

The participant was instructed to tap the two targets alternately 20 times under the following two conditions: (1) taps to any frequency (auditory signal) supplied by the frequency oscillator (each 16 frequency conditions with 0.25 Hz intervals from 0.75 to 4.5 Hz), and (2) taps at its maximum speed (free condition). The target size was 10 × 10 mm, similar to the reflective marker on the pen tip, and the distance between the targets was 240 mm, as in Woodworth’s experiment [2]. The participant did not receive any instructions regarding their body posture. The participant could self-report and had sufficient rest between the trials. Additionally, the participant was asked to self-report whether they required additional rest between trials to ensure minimal fatigue.

### 2.4. Experimental Procedure

The participant entered the laboratory, sat on the chair in front of the desk with the target, and was briefed on the task and the experiment’s risks. The participant chose to perform the task with his right hand, which is his dominant hand. The participant waited for the start of the trial with the pen tip grounded on the target on the right side as the starting position. In the 16 frequency conditions, the participant was instructed to tap into the rhythm of the frequency oscillator, listen to each frequency, and start at any given time. In the free condition, the participant was instructed to perform taps at their own maximum speed from a similar starting position. At first, 30 trials were conducted in the free condition, and 30 trials in each of the 16 frequency conditions were conducted randomly for 510 trials (30 × 17). The participant did not provide feedback on their performance until all trials were completed.

### 2.5. Data Analysis

All numerical calculations, including the analyses, were performed using Mathematica 12.3.1.0 (Wolfram Research, Champaign, IL, USA).

#### 2.5.1. Movement Frequency 

Following previous studies [12,14,16], we alternately used time-series coordinate data for the *x*-axis of the pen tip to compute the time difference between the peak (tap to the right-side target) and valley (tap to the left-side target) values. Specifically, the time difference was obtained for the number of taps. This value is called the movement time (MT). This study used MT to calculate movement frequency to examine whether the taps were conducted in the same rhythm as the frequency supplied. The calculation method divides each MT into one to calculate the movement frequency per unit of time.

#### 2.5.2. Movement Velocity and Acceleration

Velocity and acceleration were calculated by first- and second-order differentiation, respectively, using time-series coordinates in the *x*-axis direction to the direction of movement. Furthermore, both were smoothed using a 4th-order bidirectional Butterworth filter (5 Hz low pass filter). Based on previous studies [13,14,17], the highest velocity was expected to be approximately at the midpoint of the trajectory for each tap. The extremes of the velocity (positive values: movement from left to the right target, negative values: movement from right to left target) were calculated in absolute values at each movement for each trial to determine the average maximum velocity under each condition. As in previous studies, the obtained time-series data of velocity and acceleration are shown as phase diagrams of position–velocity and position–acceleration [13,14,18,19]. They were used to compare the motion control characteristics in each condition.

#### 2.5.3. Error (Distance) from Target Center Point

First, the coordinates corresponding to each trial’s tap time were calculated sequentially. The coordinate data were obtained in three dimensions; however, because the value of the *z*-axis was approximately constant when the target was reached, coordinates in two dimensions (x–y plane) were obtained for the target. The error was calculated as the distance between these coordinates from the center of the target (x–y plane). Given that the left and right targets were tapped 10 times in each trial, a total error of 20 taps per trial was obtained.

#### 2.5.4. Information Entropy

**Figure 2:**
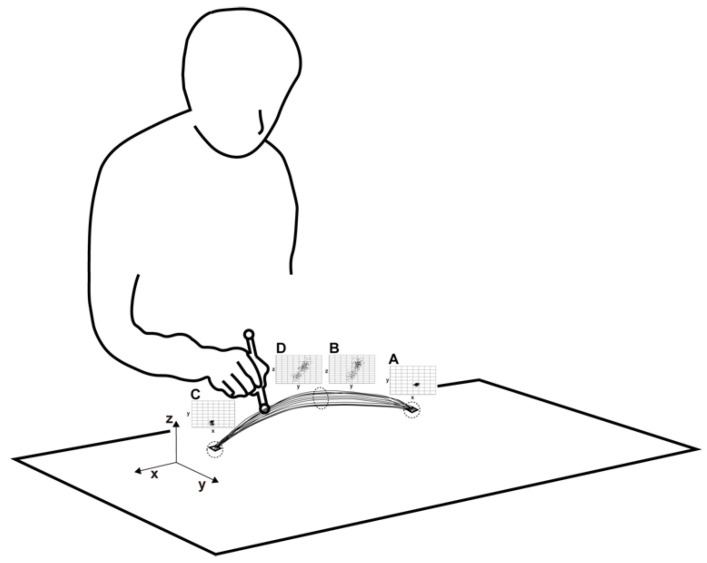
A typical example of trajectory and calculation of coordinates of each point. Each trial begins with the pen tip grounded on the right target, so the coordinates are determined at points A, B, C, D, and A in sequence. Coordinates in the x–y plane are used for A and C, the target points, and coordinates in the y–z plane for B and D, the middle points of the trajectory.

Probability is the basis of information entropy [5]. Previous studies have used the probability of data distribution at each time point of the trajectory to calculate the information entropy for discrete aiming motions [20,21,22]. A recent study showed that entropy might be calculated by separately analyzing trajectories moving in the same direction, even for recurring aiming movements such as those in this study. Further, that entropy was calculated based on the coordinates of multiple points in time from the target to the target [14]. Similar to another study [22], it was essential to consider the entropy of extreme velocity values in the direction of movement. It was found that extremes occurred at approximately 50% of the distance in the direction of movement [23]. Therefore, in this study, where the distance between targets was constant, 20 coordinates in the 2-dimensional plane (y–z plane) were calculated for each trial, particularly when passing through the 50% point (120 mm) of the distance in the direction of movement (Figure 2). These 20 points were obtained as 10 when moving from the left to the right target. Furthermore, they were acquired at 10 points, from the right to the left target. This was designated as the midpoint of the trajectory. Furthermore, the coordinates (x–y plane) of each target point and endpoint of the trajectory were used in the analysis for each of the 10 points. Information entropy was calculated at four points—the left and right target points and the two midpoints.

This study obtained the probability required to calculate information entropy following a previous study [14]. This method was based on the target size (10 mm per side). Further, entropy was calculated by encoding the coordinates of each point into a square of 10 mm per side. Multiple squares of 10 mm per side were provided on the target and midpoint planes. The probability was calculated by calculating the square of the 2-D coordinates corresponding to that point. This was calculated using $H_{1}(X)\equiv \underset{a\to 1}{\lim}H_{a}\left(X\right)=\sum Pi\log_{2}\left(1/Pi\right)$, where $P_{i}$ is the frequency distribution of data points in bin i. The limiting value of $H_{a}$ as a→1 is Shannon entropy [5,24]. Specifically, in this analysis, if all coordinates are in the same square (encoded with the same value), the value of information entropy is zero [14].

#### 2.5.5. Mutual Information

Mutual information measures the amount of information one random variable contains about another [5,25]. As in previous studies [14], this value was obtained using probabilities calculated from the coordinates of the target and midpoints. Furthermore, to consider direction, we obtained each value separately from the right target to the middle (Figure 2, A to B), center to the left target (Figure 2, B to C), left target to the middle (Figure 2, C to D), and middle to the right target (Figure 2, D to A). We averaged them (B and D) as targets in the middle to eliminate the influence of the left and right sides. Furthermore, the entropy of the previous point was compared with that of the posterior point to define relative mutual information. If entropy_before_ > entropy_after_, the mutual information is negative because that entropy is decreasing, and if entropy_before_ < entropy_after_, the mutual information is positive because that entropy is increasing.

### 2.6. Statistical Analysis

The movement frequency calculated from the MT inverse, error from the target center point, and maximum velocity of each movement were calculated as the mean and standard deviation for each condition. The information entropy shows each condition’s mean value for the two points (target and mid). The mutual information represents the value when moving from the target to the midpoint and from the midpoint to the target, considering the interpoints and directions. The values (movement frequency, error, maximum velocity) for each condition included 600 data (20 [Taps per trial] × 30 trials).

## 3. Results

### 3.1. Movement Frequency and Velocity

**Figure 3:**
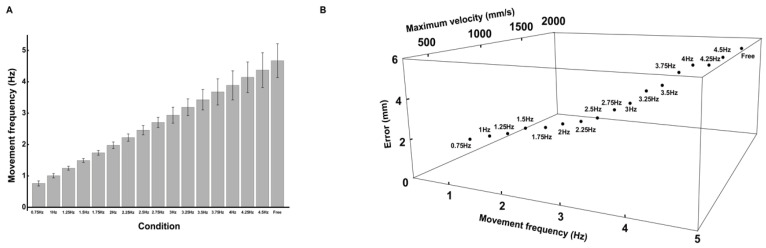
Performance variables for each condition. (**A**) Movement frequencies for each condition. (**B**) Relationship between the average movement frequency, the error from the target center point, and the average maximum velocity for each condition.

Figure 3A shows the movement frequency for each condition. The results confirm that tapping was conducted in rhythm with the frequency supplied (e.g., 1 Hz condition: 1.01 ± 0.07 Hz). Notably, in the free condition, the trials were conducted at a higher frequency than in any frequency conditions (4.67 ± 0.54 Hz).

The relationship between the average movement frequency and maximum velocity (Figure 3B): The average maximum velocity was found at the midpoint of the trajectory under all conditions, consistent with a previous study [13]. For the frequency condition, as the frequency supplied (movement frequency) increased, the average maximum velocity increased accordingly (e.g., 1 Hz condition: 690.5 ± 55.1 mm/s). The average maximum velocity for the free condition was higher than that for any frequency (1992.5 ± 74.3 mm/s).

### 3.2. Error (Distance) from Target Center Point

The relationship between the average maximum velocity and error from the target center point for each condition (Figure 3B): The results show that from 0.75 Hz to approximately 3 Hz, the error increases only slightly with increasing velocity; subsequently, the error increases with increasing velocity.

### 3.3. Information Entropy and Relative Mutual Information

**Figure 4:**
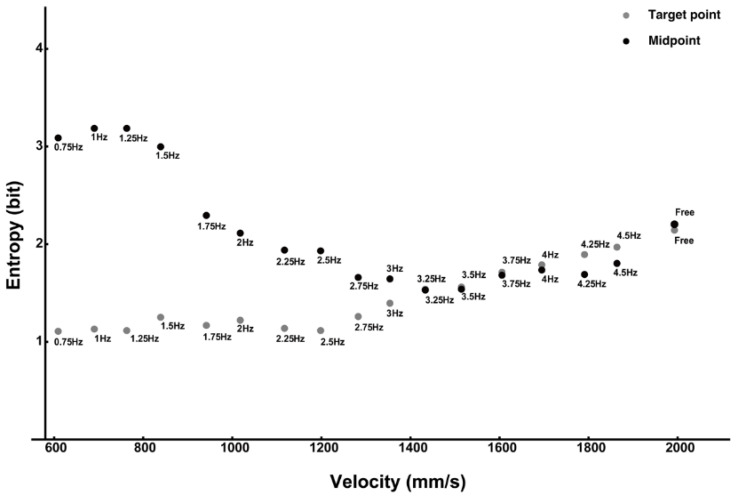
Entropy for each condition. Relationship between the average maximum velocity and the entropy at the target point for each condition (gray circle). Relationship between the average maximum velocity and the entropy at the midpoint of the trajectory for each condition (black circle).

The grey circles show the relationship between the average maximum velocity of the pen tip and entropy at the target point (Figure 4). The black circles show the relationship between the average maximum velocity of the pen tip and entropy at the midpoint of the trajectory (Figure 4). These results indicate that as the maximum velocity increases (movement frequency increases), the entropy at the target point increases, but the entropy at the midpoint of the trajectory decreases.

**Figure 5:**
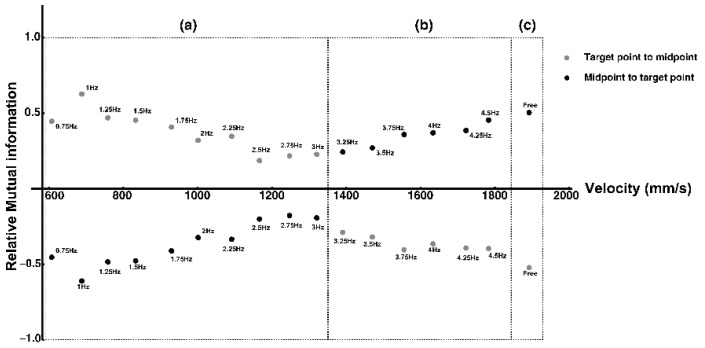
Relative Mutual information for each condition and examination of differences in information processing with changes in movement frequency. Mutual information when moved from the target point to the midpoint (gray circle) and the midpoint to the target point (black circle) for each condition. (**a**) The group associated with feedback control, where information processing occurs from the middle of the trajectory to the target (entropy decreases). (**b**) The group is associated with feedforward control, where information processing occurs (entropy decreases) from the target to the middle of the trajectory. (**c**) The group where information processing occurs stabilizes the trajectory (entropy does not change).

The gray circles show the relative mutual information moving from the target point to the midpoint for each condition (Figure 5). The trend for the frequency condition changed from positive to negative at approximately 3 Hz and 3.25 Hz. A negative proportional relationship was observed between the velocity and amount of mutual information. The black circles show relative mutual information moving from the midpoint to the target point for each condition (Figure 5). Similarly, the frequency condition trend changed from negative to positive at approximately 3 Hz and 3.25 Hz. A positive proportional relationship was observed between the velocity and amount of mutual information. The absolute values were higher under low-frequency (low velocity) and high-frequency (high velocity) conditions. The values approached zero and were lowest in the frequency range, where the positive and negative values switched. In the free condition, the two points had similar entropy values (Figure 4) and large absolute values of mutual information (Figure 5).

## 4. Discussion

This study aimed to investigate the relationship between movement speed and information processing using the following methods: introducing external control of movement speed in Woodworth’s experiment [2] to Fitts’ reciprocal tapping experiment [3], tapping the exact distance between targets at different speeds, and quantifying the process of trajectory change to investigate the relationship between the speed of motion and information processing. We used the method examined in previous studies [13,14] to examine information processing, unlike previous approaches that examined information processing based only on the variation in target endpoints. Instead, this approach considers the information entropy and mutual information, which can be calculated from the probability of multiple coordinates of multiple trajectories with arbitrary intervals between them. We determined the part of the trajectory used for information processing by examining how these values varied among the intervals.

First, the trade-off between speed and accuracy has been widely reported for the target-aiming task used in this experiment [2], and the results in Figure 3B, where the error from the target point increases as the maximum velocity increases, similarly support this relationship. Furthermore, as Woodworth [2] explained, there is a lower limit beneath which the error from the target point is not zero, regardless of the reduction in the speed of motion (Figure 3B). Woodworth explained the existence of an upper limit beyond which the error from the target point did not increase, even if the speed increased from approximately 2.3–3.3 Hz. However, in the present study, the error increased, even at frequencies above 3 Hz (Figure 3B). Although it is challenging to deduce a simple comparison because Woodworth’s experiment was not conducted above 3.3 Hz, we believe this results from the difference in the tasks. The task’s difficulty would have differed in Woodworth’s experiment, where the pen tip was moved on a flat surface. In Fitts’ experiment, height was added to the movement, and a target having a similar size to that of the pen tip was targeted.

Next, entropy was calculated using the method used in a previous study [14] by obtaining the probabilities of the variation in the coordinates at the target point and midpoint of the trajectory, in particular, where the movement velocity is considered the maximum and the control of the movement switches. Information entropy obtained using experimental data may provide a more accurate estimate of information entropy than the assumed structure of the data distribution or the entropy of an a-priori-defined task [21,22]. The results showed different relationships between the change in velocity and the magnitude of entropy at the end of the trajectory (the target point) compared to the midpoint of the trajectory. For example, as the maximum velocity (movement frequency) increased, entropy at the target point increased (Figure 4). By contrast, the entropy at the midpoint of the trajectory decreased with increasing velocity (Figure 4). The mutual information between the points is shown in Figure 5. If entropy decreases at the posterior location from the anterior location, it is shown as a negative value. The opposite is shown as a positive value. The results show two trends. First, the amount of mutual information moving from the target point to the midpoint was negatively proportional, with positive and negative values switching at 3.0 and 3.25 Hz. Second, the amount of mutual information moving from the midpoint to the target point also switched between positive and negative values at 3.0 Hz and 3.25 Hz, becoming positively proportional. We examined the relationship between movement speed and information processing based on this trend difference.

### Relation between Changes in Information Processing Points and Motor Control

Next, by considering the findings on motor control derived from the characteristic kinematic changes in many previous studies dealing with reaching motor tasks, such as Fitts or Woodworth tasks, we simultaneously examined their relationship with information processing [13,14]. In our previous studies [13,14], when the variability (distribution) of quantified values decreased toward the next point, we considered that information processing occurred because the irregularity could be regarded as reducing. This idea was based on a previously presented argument [5]. From this perspective, it can be inferred that the point on the trajectory where information processing occurred changed under low-velocity (low-movement frequency) and high-velocity (high-movement frequency) conditions.

**Figure 6:**
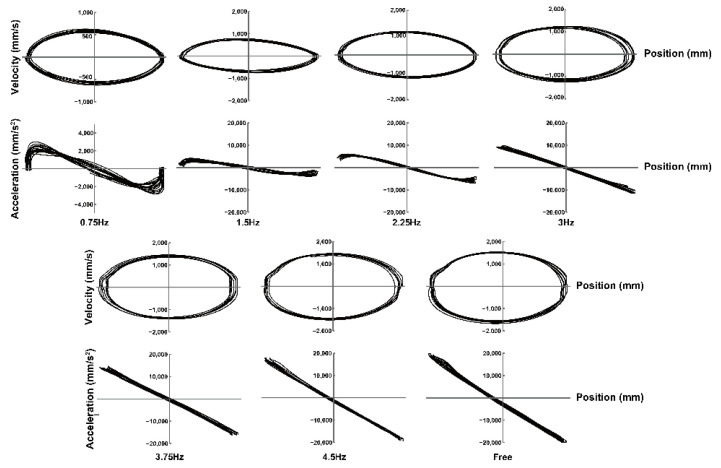
Phase diagram of typical position–velocity and phase diagram of position–acceleration for seven conditions.

First, in the low movement frequency condition group (Figure 5a), entropy decreases from the midpoint to the target point (e.g., 0.75 Hz condition, midpoint 3.09 bits, target point 1.11 bits). The position–acceleration phase diagram (Figure 6) shows that the change in acceleration near the target becomes more pronounced at lower frequencies. This represents feedback control to capture the target accurately as an N-shaped acceleration change [19,26]. Specifically, it is possible that more information processing occurs from the middle to the target point for feedback control at low movement frequencies. Furthermore, as the frequency increased, the acceleration changed from N-shaped to linear, and the amount of mutual information tended toward zero; that is, the absolute value decreased (Figure 6). Accordingly, group (a) was characterized by information processing associated with feedback control from the middle of the trajectory to the target point. However, as the movement speed gradually increases, feedback control to cope with the frequency becomes unavailable, and less information processing occurs.

By contrast, entropy increased from the midpoint to the target point in the high movement frequency condition group (Figure 5b). It decreased from the target to the midpoint (e.g., 3.75 Hz condition, midpoint 1.68-bit, target 1.47-bit). The phase diagram of position acceleration is almost a straight line in this condition group, indicating a reciprocating motion in which acceleration and deceleration are conducted continuously. It is possible that a large amount of information processing occurred from the target to the midpoint concerning the feedforward control in this group (b). Interestingly, the entropy decreased at the midpoint, even though, unlike the target point, no target must be passed through this location. This may result from information processing in the first half of the trajectory, possibly due to the unavailability of feedback control near the target. Therefore, this may reflect more information processing during the planning phase of the movement, as it was more challenging to modify the movement in response to the presented frequency than in group (a).

Third, a different trend was observed in the free condition without a supplied frequency (Figure 5c). The entropy did not increase or decrease between the points, but the amount of mutual information was relatively high. This is a curious result because information processing by the convergence of trajectories, as described above, was not performed. Nevertheless, this may reflect the result of information processing that does not expand the distribution to stabilize the trajectories. This result may be related to the findings of studies on finger tapping without movement between targets [27,28,29]. For instance, movements performed at self-generated (self-paced) frequencies are more comfortable than those performed at externally generated (externally paced) because they produce a 1/f noise. Notably, even in the sighting task performed at the self-generated maximum speed, as in the present study, the trials were conducted comfortably for the participants, suggesting that this may be related to the information processing stabilizing the trajectory. Furthermore, previous studies dealing with discrete aiming movements have shown that the proportion of pink-noise (1/f noise) structures is higher when there is no opportunity to use feedback control (i.e., when ID is low and movement frequency is high) [30,31,32]. Although these findings are not based on studies conducted at the self-generated maximum speed, as in the present study, possibly even at the maximum rate, as in the present study, different information processing could have occurred because of 1/f noise. However, the distance from the target point and the entropy of the target point in the free condition were higher than those in any other conditions, and the target was not accurately captured (Figure 4). Nevertheless, it is possible that different information processing occurred in self-generated movements compared with movements controlled by external frequencies.

Finally, a limitation of this study was that the characteristics of movement speed and information processing discussed above were obtained from a single participant. We cannot deny the possibility that the results are specific to this participant. Therefore, increasing the number of participants and examining these characteristics’ generality is necessary. The differences between self-paced and externally paced information processing should be investigated further.

## 5. Conclusions

This study added Woodworth’s control of the speed of movement to Fitts’ experimental paradigm to quantify trajectory variation during the continuous aiming task as a value of information entropy and mutual information and to estimate the amount of information processing. The average maximum velocity also increased with an increase in movement frequency. We found that the point at which information processing considered from the convergence of trajectories changes from the second half of the trajectory, associated with feedback control, to the first half, associated with feedforward control at approximately 3 Hz. Furthermore, the self-paced maximum velocity condition indicated that information processing might have been performed that did not widen the trajectory distribution, that is, stabilized the trajectory.

## Data Availability

The datasets generated during and/or analyzed during the current study are available from the corresponding author on reasonable request.
